# Peer Support in Online Women’s Health Communities: Mixed Methods Formative Analysis of Reddit Discourse

**DOI:** 10.2196/87782

**Published:** 2026-05-04

**Authors:** Kimia Tuz Zaman, Wordh Ul Hasan, Nova Ahmed, Juan Li

**Affiliations:** 1 Department of Computer Science, North Dakota State University, Quentin Burdick Building Room 258, 1320 Albrecht Boulevard, Fargo, ND, 58105, United States, 1 701-231-9662; 2 Department of Math Science & Technology, University of Minnesota Crookston, Crookston, MN, United States; 3 Department of Computer Science, Tuskegee University, Tuskegee, AL, United States; 4 Department of Electrical and Computer Engineering, North South University, Dhaka, Bangladesh

**Keywords:** women’s health, peer support, online health communities, formative research, systemic clinical dismissal, cultural competency, social support

## Abstract

**Background:**

Stigmatized women’s health issues, such as polycystic ovary syndrome (PCOS) and endometriosis, are often marginalized or dismissed in traditional clinical settings. This drives individuals to seek peer support in anonymous online communities such as Reddit. While these digital platforms host critical discussions, they are often designed as static information repositories, failing to account for the complex emotional, temporal, and cultural dynamics that shape users’ support needs. There is a disconnect between the lived experiences of users—particularly feelings of clinical dismissal and the need for culturally specific advice—and the design of the sociotechnical systems they rely on.

**Objective:**

This study aimed to deconstruct support practices in online women’s health forums to provide a formative basis for designing more responsive digital health systems. We analyzed the intersections of discussion topics, emotional expression, temporal shifts (specifically the impact of the COVID-19 pandemic), and culturally situated discourse to identify unmet user needs and effective peer-support patterns.

**Methods:**

We conducted a large-scale, mixed-methods analysis of 4995 posts and 460,317 comments from 5 major women’s health subreddits (r/WomensHealth, r/TwoXChromosomes, r/BirthControl, r/Endometriosis, and r/PCOS). Computational methods included Latent Dirichlet Allocation for topic modeling, Valence Aware Dictionary for Sentiment Reasoning for sentiment analysis, and the NRC Emotion Lexicon for granular emotion classification. We segmented the data into pre-, during-, and post–COVID-19 periods to analyze temporal shifts. This quantitative analysis was complemented by a 2-phase qualitative thematic analysis to identify and characterize engagement patterns within 147 validated culturally situated threads.

**Results:**

Our analysis revealed that the most prevalent and emotionally negative topic was “Pain & Doctor Visits,” which was uniquely characterized by high levels of fear and sadness linked to systemic clinical dismissal. The COVID-19 pandemic triggered a significant topical “turn inward,” with discussions shifting away from social or political issues and toward somatic concerns (eg, “PCOS” “Pain & Doctor Visits”). Paradoxically, this period saw a simultaneous rise in both negative emotions (eg, fear and sadness) and expressions of community trust. Critically, our qualitative analysis of culturally situated discourse uncovered a consistent three-stage “playbook” for effective support: (1) *Affirmation* to establish psychological safety and validate cultural experiences; (2) *Information Scaffolding* to provide actionable, culturally tailored advice; and (3) *Intercultural Bridging* to facilitate community-wide learning and empathy.

**Conclusions:**

Online health forums operate as essential, resilient sociotechnical infrastructures that actively compensate for failures and gaps in formal health care. The “Affirmation-Scaffolding-Bridging” model identified in our research provides a clear, formative framework for designing future digital health interventions. These findings can guide the development of new platforms that are emotionally aware, culturally responsive, and adaptive to user needs and external crises.

## Introduction

Digital platforms have emerged as critical sociotechnical systems for health support, yet they are often designed without a deep understanding of the sensitive, stigmatized, and emotionally charged topics they host [1,2]. Concerns related to reproductive, menstrual, and intimate health—particularly conditions such as polycystic ovary syndrome (PCOS), endometriosis, and perimenopausal symptoms—are routinely marginalized due to stigma, cultural taboos, systemic misinformation, and medical dismissal [3]. These conditions remain undiagnosed or are mismanaged, exacerbating frustration and anxiety among those affected [4]. For example, women with PCOS engage in self-experimentation and support-seeking practices to manage uncertainty and invisibility in traditional care settings [5]. Endometriosis is a condition in which endometrial-like tissue grows outside the uterus, often causing chronic pelvic pain and infertility, and patients frequently become self-advocates, leveraging independent research to navigate systemic obscurity, stigma, and clinical ambiguity [6]. Women’s health issues are further complicated for individuals from marginalized or culturally diverse backgrounds, who face compounded barriers of cultural misunderstanding and access inequity [7]. Although communities within sociotechnical platforms such as Reddit and FemTech apps offer potential spaces for solidarity and knowledge exchange [4], the design of these technologies often fails to account for emotional labor, culturally specific expression, and shifting real-world contexts such as medical crises or policy disruptions [8]. The central challenge is therefore not merely to create health support platforms but to design systems that are emotionally responsive, culturally situated, and resilient—capable of scaffolding advocacy, sustaining cultural expression, and adapting to collective disruptions.

Prior research has established that online health communities can profoundly support emotional expression, peer connection, and shared learning, especially when clinical systems fall short. For instance, platforms such as CaringBridge illustrate how expressive writing correlates with greater user engagement and well-being outcomes [9]. Studies in mental health communities likewise highlight that posts imbued with emotional expression and social experience attract more engagement and resonate deeply with peer networks [10]. Anonymity also emerges as a vital design affordance—relieved from stigma, users seek and offer emotional support more freely [11]. Meanwhile, feminist scholars critique dominant narratives around women’s bodies and health, arguing for more inclusive and intersectional design approaches [12]. These efforts collectively demonstrate growing sensitivity to emotion, identity, and context.

Despite these insights, existing work remains compartmentalized. Few studies examine how emotional tone, cultural specificity, and temporal dynamics—such as shifts catalyzed by crises or societal changes—interact to shape user experience. Temporality matters: sudden events such as pandemics alter what health topics dominate discussion and how users emotionally engage. Culture matters: taboos and norms vary widely across communities, influencing what is shared, how it is interpreted, and what support is desired. And yet, these dimensions are rarely studied in conjunction. There is a notable gap in knowledge: we lack holistic, empirical understanding of how emotion, temporality, and culture intersect within women’s health communities to influence support-seeking, discourse dynamics, and platform engagement. Addressing this gap is essential if we hope to design digital systems that responsively scaffold culturally situated, emotionally resonant, and adaptively evolving support ecosystems.

To address this gap, we conducted a large-scale, mixed methods analysis of women’s health discourse on Reddit, examining 4995 posts and more than 460,000 comments across 5 major women’s health subreddits. Our methodological approach combined computational techniques—Latent Dirichlet Allocation (LDA) for topic modeling, Valence Aware Dictionary for Sentiment Reasoning (VADER) for sentiment analysis, and the NRC Emotion Lexicon for emotion classification—with a temporal segmentation of discussions across pre–, during, and post–COVID-19 periods. To capture the role of cultural context, we implemented a 2-phase analysis: first, a computational filtering of culture-sensitive posts using keyword detection; second, a qualitative thematic analysis of this subset to characterize engagement patterns around Affirmation, Information Scaffolding, and Intercultural Bridging. This hybrid approach reflects a tradition of blending large-scale computational methods with qualitative depth to unpack the nuances of online health discourse [4,9]. By integrating multiple lenses—topics, emotion, temporality, and culture—our study seeks to provide a holistic account of how women negotiate support, advocacy, and identity in digital spaces, and to surface design opportunities for sociotechnical systems that are more emotionally responsive, culturally adaptive, and resilient to disruption.

Guided by this multidimensional approach, our investigation centers on 4 research questions (RQs) that unpack the intersections of content, emotion, temporality, and culture in online women’s health discourse. These questions allow us to move beyond narrow disease-specific studies and toward a holistic understanding of how digital platforms mediate support-seeking across diverse contexts:

RQ1: What are the prevalent topics and themes in naturally occurring online discussions within women’s health support communities?

RQ2: How do individuals express emotions and sentiments in these discussions, and what role do these expressions play in shaping social support dynamics?

RQ3: How have patterns of support-seeking evolved across the pre–COVID-19, during COVID-19, and post–COVID-19 periods?

RQ4: To what extent do cultural factors and sensitivities influence discourse and engagement within online women’s health communities?

## Methods

### Data Collection

In our pursuit to examine online communities dedicated to women’s health, we focused on the social media platform Reddit. Known for its expansive “subreddits” and the relative anonymity it grants its users, Reddit provides an ideal environment for open, candid discussions on sensitive health topics. We carefully selected 5 subreddits—r/WomensHealth, r/TwoXChromosomes, r/BirthControl, r/Endometriosis, and r/PCOS—due to their active participation, large and engaged user bases, and clear alignment with our research objectives. These subreddits encompass a wide array of women’s health concerns, including general wellness (r/WomensHealth), social and personal topics from a female perspective (r/TwoXChromosomes), contraceptive methods (r/BirthControl), and condition-specific communities focusing on endometriosis (r/Endometriosis) and polycystic ovary syndrome (r/PCOS). By concentrating on these subreddits, we aimed to capture a selection of high-engagement conversations reflective of different health challenges and support-seeking behaviors. We acknowledge that this selection does not represent the entirety of women’s health on the platform. However, by including both general and condition-specific forums, this selection provides a robust representation of a wide range of prevalent health challenges and support-seeking behaviors.

While other platforms (eg, Facebook groups and specialized health forums) also host women’s health discussions, Reddit’s design facilitates high volumes of user-generated content and community-driven threads. The added benefit of anonymity on Reddit often encourages more open sharing of personal experiences, which is critical when exploring sensitive health topics. Furthermore, these selected subreddits offered consistent, ongoing discussions, making them ideal for studying both historical and current trends in women’s health support-seeking.

To collect the data, we used a custom Python script leveraging the Python Reddit Application Programming Interface (API) Wrapper. Our script connected to the Reddit API using valid credentials and systematically retrieved the top 1000 posts (based on upvotes) from each of the 5 subreddits. We applied the time_filter = “all” parameter—which instructs Python Reddit API Wrapper to rank posts by their cumulative, all-time upvote totals rather than any fixed recency window—to encompass the entire available posting history of each community, thus capturing discussions spanning from each subreddit’s inception up to the time of data collection. This sampling strategy was chosen deliberately to capture the most resonant, community-validated discourse, as upvotes serve as a primary indicator of posts that the community deems significant, relevant, or impactful. While this approach systematically focuses on high-engagement content, it is aligned with our goal of analyzing the prevalent themes and support dynamics that have the most visibility and influence within these communities. We also acknowledge that this “’top post”’ method biases our dataset toward high-visibility content and may not capture the full spectrum of less-upvoted or more niche discussions, which we address in our limitations section. Furthermore, by sampling the top posts from each subreddit independently, we used a stratified approach to ensure that smaller, specialized forums were not underrepresented in the final dataset. Alongside the main submissions, we also gathered all comments nested under each retrieved post to gain deeper insights into the nuances of community conversations. This yielded a significantly larger volume of comment-level data, enabling a more comprehensive understanding of participant interactions and support-seeking dynamics. Upon collection, each post and comment was time-stamped and stored along with metadata (such as author ID, score, and number of comments) in Excel spreadsheets. We included only English language posts and comments to maintain linguistic consistency for subsequent text analyses.

In total, our data collection yielded 4995 posts and 460,317 comments across the 5 subreddits. The combination of these communities and the breadth of user engagement in them provided a rich dataset for examining how women seek and offer support on topics such as contraception, chronic gynecological conditions, general women’s health, and broader social issues. While we acknowledge the value of exploring additional women’s health groups on other platforms (eg, Facebook and dedicated health forums), Reddit’s ease of data access, extensive public discussions, and built-in anonymity made it the most suitable primary source for this study.

### Topic Modeling (LDA)

To uncover latent thematic structures in the discussions, we used LDA topic modeling. LDA is a generative probabilistic model well suited for discovering hidden topics in large collections of texts without supervision. We chose LDA to summarize the vast and unstructured posts into interpretable themes, enabling us to address RQ1 about prevalent topics. After experimenting with several topic counts, we set the number of topics to 15, balancing granularity with interpretability. We determined the optimal number of topics (*k*=15) through an iterative process involving both quantitative metrics and qualitative validation. We systematically evaluated LDA models with *k* ranging from 5 to 25 topics using coherence score analysis (c_v metric). Quantitative evaluation revealed that *k*=15 achieved the highest coherence score (0.4795), outperforming both lower values (*k*=8: 0.4513, *k*=10: 0.4377) and higher values (*k*=16: 0.4638, *k*=20: 0.4516). While neighboring configurations (*k*=13‐14, *k*=21‐23) showed comparable coherence, qualitative inspection confirmed *k*=15 as optimal. Models with fewer topics (*k*<15) conflated distinct health conditions—for instance, merging endometriosis-specific discussions with general pain narratives. Conversely, models with more topics (*k*>15) exhibited redundant fragmentation, such as splitting birth control discussions into marginally distinct subtopics that lacked clinical or thematic differentiation. Thus, *k*=15 balanced quantitative coherence with qualitative interpretability, yielding semantically distinct topics without overfragmentation. We used each post (combined title + body) as a document in the LDA model (thus 4995 documents). The LDA algorithm was implemented using scikit-learn’s LDA with the following parameters: n_components=15, max_iter=50 (maximum passes over the corpus), max_doc_update_iter=400 (per-document variational inference steps per pass), learning_method=“batch” (full corpus updates per iteration), and random_state=42 for reproducibility. Symmetric priors were used for both the document-topic (α) and topic-word (*β*) distributions, consistent with scikit-learn defaults. Convergence was monitored by computing held out perplexity every 5 passes using evaluate_every=5, with a tolerance threshold of 0.1. Text preprocessing applied a document frequency filter (max_df=0.95, min_df=2) and a combined stopword list merging Natural Language Toolkit’s English stopwords with 15 domain-specific terms (eg, “feel,” “know,” and “really”) to reduce noise from conversational filler. Each post was then assigned a dominant topic (the topic with highest probability for that post) to facilitate subsequent quantitative comparisons (eg, topic frequency and temporal trends). LDA was appropriate here because it can capture the mixture of topics in user-generated text and has been used successfully in similar health forum analyses. The resulting topics were interpreted and labeled based on their top keywords and a review of representative posts per topic.

### Sentiment and Emotion Analysis

We analyzed the overall sentiment of each post using VADER, a lexicon and rule-based sentiment analysis tool designed for social media content. VADER yields a compound score (range –1 to +1) that represents the overall sentiment polarity, as well as proportions of negative, neutral, and positive words. We selected VADER because it is tailored to informal text and captures context such as negations and emoticons, making it well suited for Reddit posts. This allowed us to quantify the emotional valence of discussions (RQ2) in a replicable way. Each post’s text was fed into VADER to compute sentiment metrics. We interpreted the compound score as an indicator of the post’s overall emotional tone (positive vs negative), while also considering the balance of positive or negative word usage (eg, a post might contain both reassurance and anxiety). Using VADER (a lightweight lexicon method) on thousands of posts was efficient and ensured consistency, complementing qualitative interpretations of community tone. Prior research has shown that such sentiment analysis can illuminate how affect is expressed in peer support contexts.

In addition to sentiment polarity, we performed a more granular emotion analysis using the NRC Emotion Lexicon (also known as NRCLex). The NRC lexicon maps English words to 8 basic emotions: anger, anticipation, disgust, fear, joy, sadness, surprise, and trust. We chose this approach to capture the multidimensional emotional expressions in posts (beyond just “positive” or “negative”), addressing RQ2’s focus on how specific emotions play a role in the discourse. For each post, we computed the frequency of words associated with each of the 8 emotions using the NRC lexicon. These frequencies were normalized by post length to obtain comparable emotion scores (approximately the percentage of words in the post conveying each emotion). The result was an emotion profile for every post (and by extension, for each topic and time period, by aggregating posts accordingly). The NRC method allowed us to identify, for example, whether discussions on certain topics were characterized by high fear (eg, anxiety about symptoms) or high trust (eg, gratitude toward the community). Using a lexicon-based approach ensured transparency in how emotions were identified, and although it may miss sarcasm or context-specific emotion expression, it provided a broad overview of emotional tones in a large dataset. The NRC Emotion Lexicon, while standard in the field, carries a known limitation: it assigns emotion labels based on the connotative associations of words out of context, which can result in misclassification in specialized discourse—a bias that has been documented and empirically corrected in prior work [1]. Importantly, this limitation does not alter the core interpretive validity of our findings. Lexicon-based approaches trade some context sensitivity for domain independence and scalability, making them well suited for large-scale comparative analyses across topics and time periods [7]. Critically, in our study the NRC was applied consistently across all topics and COVID-19 periods; therefore, any systematic misclassification would affect all groups equally, preserving the validity of the relative comparisons that form the basis of our interpretations—such as the difference in fear and sadness scores between “Pain & Doctor Visits” and “Weight Check-Ins,” or the rise in trust scores across COVID-19 periods. We address this constraint explicitly in our “Limitations” section and recommend that future work use context-aware large language models to validate lexicon-assigned emotion scores in clinical text.

We implemented these analyses using the nltk.sentiment.vader library for sentiment and the NRCLex library for emotion classification. For VADER, we used the compound score as a continuous variable (–1 to +1) rather than binning posts into binary positive or negative categories, preserving the nuance of mixed-sentiment posts. For the NRC analysis, we treated emotional states as non–mutually exclusive vectors. Since a single post can simultaneously express multiple emotions (eg, “Fear” regarding a diagnosis and “Trust” in the community), we calculated the frequency density of each emotion independently. We did not force a “dominant” emotion label per post; instead, we generated an 8-dimensional emotion profile for each topic, allowing us to capture complex emotional co-occurrences without artificial conflict resolution.

### COVID-19 Period Segmentation

To examine the impact of the COVID-19 pandemic on community discussions (RQ3), we segmented the dataset into 3 time periods: pre–COVID-19 (posts created before January 1, 2020), during COVID-19 (posts from January 1, 2020, through December 31, 2021), and post–COVID-19 (posts from January 1, 2022, onward). This roughly corresponds to the prepandemic era, the initial pandemic outbreak and peak disruption period, and the period following widespread vaccination and the easing of lockdowns. We tagged each post with its respective period based on the time stamp. This segmentation allowed for a comparative analysis of topic prevalences and emotional patterns across these distinct phases of the pandemic. By counting posts per topic in each period and computing the percentage of posts devoted to each topic, we could observe shifts in community focus over time. We also aggregated the NRC emotion scores by period to detect changes in overall emotional expression (eg, whether fear words were more common during the pandemic). Statistical testing was then applied to assess the significance of observed changes: we used a chi-square test of independence to examine whether the distribution of topics differed by period (contingency table of topic vs period), and we used ANOVA to compare sentiment or emotion means across periods. This mixed quantitative approach ensures that any reported differences in RQ3 are robust and not due to random variation.

### Identifying and Analyzing Culturally Situated Discourse

#### Overview

To address RQ4 on how cultural factors shape women’s health discussions and engagement, we used a 2-phase, mixed methods approach designed to move beyond simple keyword spotting and capture the nuance of culturally situated discourse and response patterns.

#### Phase 1: Computational Filtering

We first screened the full corpus using a curated lexicon of culture-related terms spanning (1) *religious contexts* (eg, Muslim, Islam, Ramadan, Catholic, Jewish, Shabbat, Hindu, and Temple), (2) *ethnic or national descriptors* (eg, Asian, South Asian, Latina, Black, Indigenous, Middle Eastern, Arab, and Persian), and (3) *cultural concepts* relevant to women’s health (eg, tradition, taboo, modesty, stigma, family pressure, conservative, immigrant, and first generation).

- *Procedure*: For each post, we concatenated title and body text, lowercased, and applied word-boundary matching (regex) to reduce false positives due to substring overlaps (eg, “ramadan” vs “dramatically”).
- *Flagging rule*: A post was flagged as culture-sensitive if it contained ≥1 lexicon term.
- *Outcome*: This filtering yielded a candidate set of 335 posts for qualitative analysis.

This conservative identification step prioritized *recall* (capturing a broad candidate pool), with subsequent human coding used to validate cultural situatedness and characterize engagement.

#### Phase 2: Human Qualitative Thematic Analysis

Two researchers with human-computer interaction (HCI) training (KTZ and WUH) independently coded the *335* flagged posts and their comment threads in a structured, 2-step process. We first validated whether each flagged post indeed discussed a women’s health issue through an explicit cultural lens. A post was confirmed *culturally situated* if it (1) directly referenced cultural/religious/ethnic contexts and (2) connected those contexts to the health issue (eg, cultural taboos affecting care-seeking, religious practices shaping symptom management, and family or community norms influencing contraceptive decision-making). Incidental mentions (eg, a nationality in a travel anecdote without implications for the health concern) were not considered culturally situated.

The coders adopted a consensus-based, iterative approach to codebook development. We began by jointly drafting an initial rubric based on 10% of the sample (n=34 posts), refining inclusion or exclusion criteria through open coding. Interrater reliability was assessed on this development subsample (Cohen κ=0.952), indicating strong agreement. To maintain this rigor across the full dataset, we used a “negotiated agreement” protocol rather than simple independent coding. The 2 researchers (WUH and KTZ) coded the remaining dataset independently but flagged ambiguous or borderline cases for review. These flagged items were resolved during weekly consensus meetings, where the researchers discussed the specific context until 100% agreement was reached. This process prevented “coder drift” and ensured that complex cultural nuances were interpreted consistently throughout the analysis.

For each post validated as culturally situated by both coders, we analyzed *the top 10 most upvoted comments* (when available), excluding deleted or bot entries, to characterize how the community responded. Engagement was coded using three a priori categories derived from prior work on peer support [8] and refined during pilot coding: (1) *Affirmation*: validating culturally specific experiences (eg, “I went through the same during Ramadan—you’re not alone”); (2) *Information Scaffolding*: culturally tailored advice or resources (eg, dietary guidance compatible with fasting, and directories for language-concordant or culturally sensitive clinicians); and (3) *Intercultural Bridging*: respectful cross-cultural dialogue that signals learning or allyship (eg, questions from users outside the culture seeking to understand practices and be supportive).

Comments that did not fit these categories (eg, generic tips without cultural relevance) were labeled “Other.” Coders recorded the primary engagement type per comment, and we maintained an audit trail of decision notes to support transparency. The complete codebook is provided in Multimedia Appendix 1.

This 2-phase design aligns with mixed methods best practices in HCI: computational scale is used to surface a broad candidate set, and human qualitative depth validates cultural situatedness and explicates *how* support unfolds. The approach yields a defensible basis for quantifying engagement patterns while preserving the contextual nuance necessary to inform culturally responsive design.

### Ethical Considerations

This study involved a secondary analysis of existing data obtained exclusively from publicly accessible Reddit posts, with no direct interaction or intervention involving human participants. In accordance with the North Dakota State University Human Research Protection Program, Special Research Topics, Section 11.3: Secondary Analysis of Existing Data, this project was evaluated under the criteria for research not subject to institutional review board (IRB) oversight. Specifically, consistent with Policy 2.4 (Information in the Public Domain), research using publicly available information does not require IRB review when researchers have no interaction with individuals and the data are not restricted by privacy or confidentiality protections. The Reddit data analyzed were publicly posted prior to the initiation of this study and were originally generated for purposes unrelated to the current research. The research team did not obtain, access, or record any direct or indirect identifiers; did not attempt to contact users; and had no access to any linkage keys that could enable identification of individuals. As such, the study did not constitute North Dakota State University’s engagement in human subjects research and did not require IRB review, exemption determination, or a protocol number. Despite the public nature of the data, the study adhered to established ethical guidelines for internet research. All data were systematically deidentified prior to analysis, including the removal of usernames, pseudonyms, and any potentially identifying metadata, to further protect user privacy and minimize risks of individual or group harm.

### Researcher Positionality

The research team approaches this study through an interdisciplinary lens that bridges HCI, computer science, and artificial intelligence, integrating perspectives from both the Global South and North America. This geographic and disciplinary diversity was central to our analytical framework, allowing us to balance technical rigor with cultural sensitivity. Our team combines expertise in developing patient-centric artificial intelligence systems and predictive health models with a critical grounding in feminist HCI and the sociotechnical analysis of online support communities. This dual focus influenced our decision to adopt a mixed methods approach: we validated our computational pipelines (LDA, VADER, and NRC) through a qualitative coding protocol that prioritized “negotiated agreement” to check individual biases. We approached the data not merely as text to be mined but as sensitive narratives embedded in complex social structures, remaining acutely aware of our power as researchers interpreting vulnerable health disclosures and striving to represent cultural nuances with empathy and context.

## Results

### Overview

This section presents the findings of our analysis, organized sequentially by the 4 RQs. We first report the prevalent topics (RQ1); then examine sentiment and emotion (RQ2); trace how these patterns evolve across pre–, during, and post–COVID-19 periods (RQ3); and finally analyze how cultural sensitivity shapes community engagement (RQ4).

### RQ1: Prevalent Topics and Themes

The LDA analysis identified 15 distinct topics within the dataset, detailed in Table 1. These topics encompass both core, recurring health concerns and event-driven themes related to external factors. The analysis revealed that the most frequent discussions did not center on routine health questions but rather on systemic issues and chronic management. The 3 most prevalent topics were “Pain & Doctor Visits” (n=786), “Birth Control Methods” (n=708), and “PCOS & Weight/Body” (n=548).

**Table 1.** Summary of the 15 topics identified by Latent Dirichlet Allocation, with post counts and top representative keywords.

Topic name	Post count	Top keywords
Pain & Doctor Visits	786	pain, doctor, told, said, went
Birth Control Methods	708	iud, period, control, birth, pill
PCOS^a^ & Weight/Body	548	pcos, weight, people, want, body
Men/Women Interactions	447	said, told, man, time, asked
Sex & Pregnancy	379	sex, want, pregnant, control, time
Abortion & Rights	354	abortion, women, life, state, rights
Body Image & Hair	328	hair, women, people, men, body
Daily Life (Endo)	322	endo, pain, endometriosis, time, edit
Endometriosis	208	endo, endometriosis, years, told, people
Doctor & BC^b^ Discussions	203	birth, control, doctor, women, pills
PCOS & Fertility	220	pcos, pregnant, months, test, insulin
PCOS Diet & Insulin	144	eat, weight, day, eating, insulin
Weight Check-Ins	160	thank, lbs, weight, post, women
PCOS & Healthcare Access	117	pcos, health, need, doctors, insurance
COVID/Vaccine Research	71	vaccine, clinical, smell, trial, study

a PCOS: polycystic ovary syndrome.

b BC: birth control.

The most prevalent topic was “Pain & Doctor Visits” (786 posts). A qualitative review of these posts indicates that the discourse focuses heavily on the process of seeking care, rather than specific disease symptoms alone. Narratives in this category frequently describe interactions with health care providers, including experiences of diagnosis, communication challenges, and managing dismissal in clinical settings.

“Birth Control Methods” (708 posts) emerged as the second most frequent topic. Discussion threads often involve users reconciling medical guidance with their subjective physical experiences. For instance, one user described the difficulty of interpreting symptoms that contradicted expected clinical outcomes: “Since being on the coil, my periods have completely stopped, but I still occasionally get all the period symptoms (painful boobs, bloating, cramps, icky skin and oily sweat)—just no bleeding. This is driving me nuts, as it just feels wrong somehow...” This quote illustrates a recurring pattern where users leverage the forum to validate embodied knowledge that aligns imperfectly with prescribed medical narratives .

The topic “PCOS & Weight/Body” (548 posts) focused on the management of chronic conditions. Unlike acute health inquiries, these posts typically address interconnected, long-term challenges, reflecting the continuous nature of managing the condition outside of episodic clinical visits.

Finally, the “Abortion & Rights” topic (354 posts) demonstrated a distinct lexical signature that juxtaposes clinical terms (“pregnant” and “care”) with political vocabulary (“rights,” “state,” “law,” and “vote”). This analysis identifies the topic as a space where personal health concerns intersect directly with political and legal discourse, differentiating it from strictly biomedical discussions within the forum.

### RQ2: Sentiment and Emotion

#### Sentiment Patterns Across Topics

**Figure 1.**
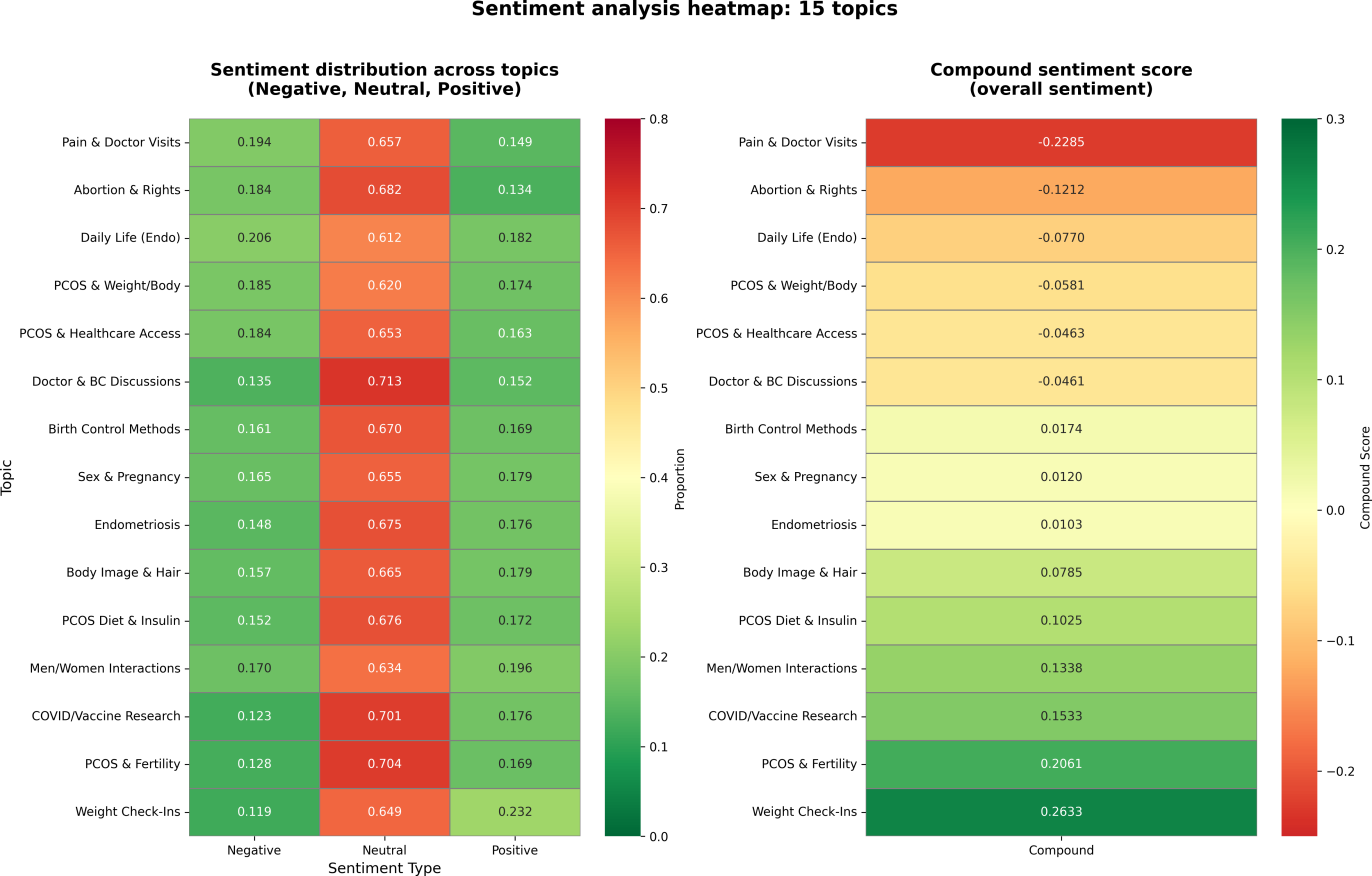
Heatmap of topic-wise sentiment (negative/neutral/positive/compound) using Valence Aware Dictionary for sEntiment Reasoning. Darker cells indicate higher values. Pain & Doctor Visits is most negative overall, Weight Check-Ins is most positive, and information-forward topics (eg, Doctor & BC Discussions) are predominantly neutral. BC: birth control; PCOS: polycystic ovary syndrome.

The VADER sentiment analysis revealed distinct variations in emotional tone across the 15 identified topics, as illustrated in the heatmap in Figure 1 and detailed in Table 2. The analysis shows a stratification of sentiment, ranging from highly negative compound scores in topics related to clinical friction to positive scores in topics focused on peer support and personal progress.

**Table 2.** Topic-wise sentiment analysis results using Valence Aware Dictionary for Sentiment Reasoning^a^.

Topic name	Negative	Neutral	Positive	Compound
Pain & Doctor Visits	0.1940	0.6566	0.1493	−0.2285
Abortion & Rights	0.1842	0.6821	0.1337	−0.1212
Daily Life (Endo)	0.2059	0.6120	0.1821	−0.0770
PCOS^b^ & Weight/Body	0.1855	0.6204	0.1740	−0.0581
PCOS & Healthcare Access	0.1838	0.6532	0.1629	−0.0463
Doctor & BC Discussions	0.1346	0.7131	0.1523	−0.0461
Birth Control Methods	0.1610	0.6701	0.1689	0.0174
Sex & Pregnancy	0.1651	0.6554	0.1795	0.0120
Endometriosis.	0.1482	0.6753	0.1765	0.0103
Body Image & Hair	0.1567	0.6646	0.1786	0.0785
PCOS Diet & Insulin	0.1520	0.6765	0.1715	0.1025
Men/Women Interactions	0.1697	0.6344	0.1959	0.1338
COVID/Vaccine Research	0.1235	0.7009	0.1756	0.1533
PCOS & Fertility	0.1278	0.7036	0.1686	0.2061
Weight Check-Ins	0.1192	0.6486	0.2322	0.2633

a Shown are the average negative, neutral, and positive sentiment proportions, and the average compound sentiment score for posts classified under each topic (topics 0‐14). The compound score ranges from –1 (most negative) to +1 (most positive), summarizing the overall sentiment of the topic’s posts.

b PCOS: polycystic ovary syndrome.

As detailed in Table 2, topics centered on information exchange, such as “Doctor & BC Discussions,” were characterized by high levels of neutral sentiment (0.713). In contrast, “PCOS & Weight/Body” exhibited a mixed sentiment profile with a compound score near zero (−0.0581). Qualitative analysis indicates that this reflects the conflicting emotions often associated with diagnosis. For instance, users frequently expressed simultaneous relief and pain, as illustrated by one post: “...on one hand it’s nice to finally have an answer...but at the same time knowing that you have it and you can’t do much about it hurts.” The text also highlighted interpersonal impacts, with users noting the “burden in our lives” regarding the strain on relationships.

The topic “Pain & Doctor Visits” registered the lowest compound sentiment score in the dataset (−0.2285), significantly lower than all other categories (Table 2). This quantitative finding aligns with the qualitative themes identified in RQ1. Textual analysis indicates that this negativity is largely driven by narratives regarding clinical dismissal and invalidation, rather than descriptions of physical symptoms alone.

Similarly, the “Abortion & Rights” topic recorded a notably negative compound sentiment score (−0.1212). While this topic involves political themes, the sentiment analysis indicates a predominance of distress. Qualitative review of these threads suggests that the negative sentiment is primarily driven by expressions of anxiety and fear regarding access to care and legal uncertainty, rather than abstract political debate or righteous anger.

In contrast to the negative sentiment observed in clinical topics, the analysis identified clusters of positive sentiment. The topics “Weight Check-Ins” and “PCOS & Fertility” recorded the highest compound scores in the dataset (+0.2633 and +0.2061, respectively). Qualitative review indicates that these discussions are characterized by mutual encouragement and the sharing of personal milestones, functioning primarily as venues for peer validation and optimism.

#### Emotional Signatures of Different Topics

**Figure 2.**
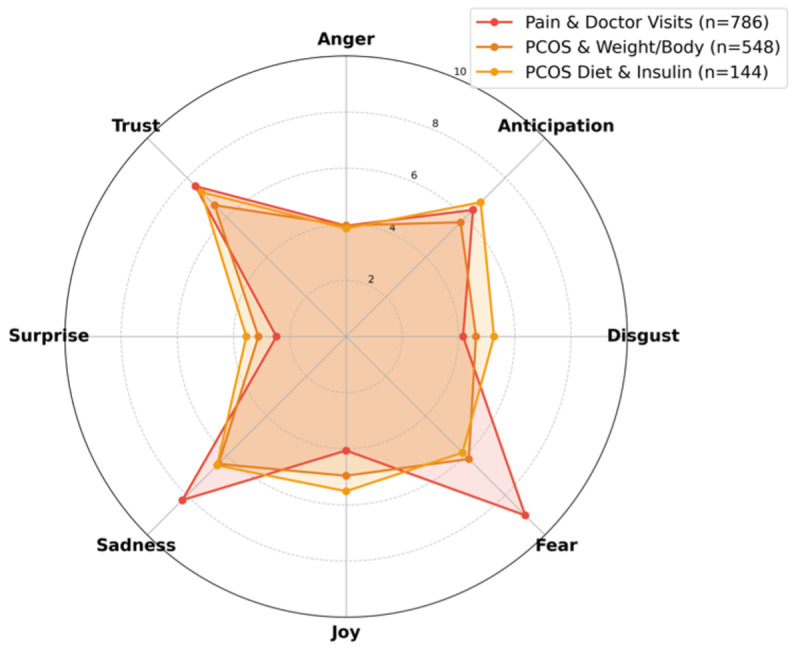
High emotional intensity topics. Radar of 8 NRC emotions for Pain & Doctor Visits (n=786), PCOS & Weight/Body (n=548), and PCOS Diet & Insulin (n=144). Pain & Doctor Visits peaks on Fear/Sadness; PCOS clusters mix higher Anger/Disgust/Sadness with relatively high Trust. PCOS: polycystic ovary syndrome.

**Figure 3.**
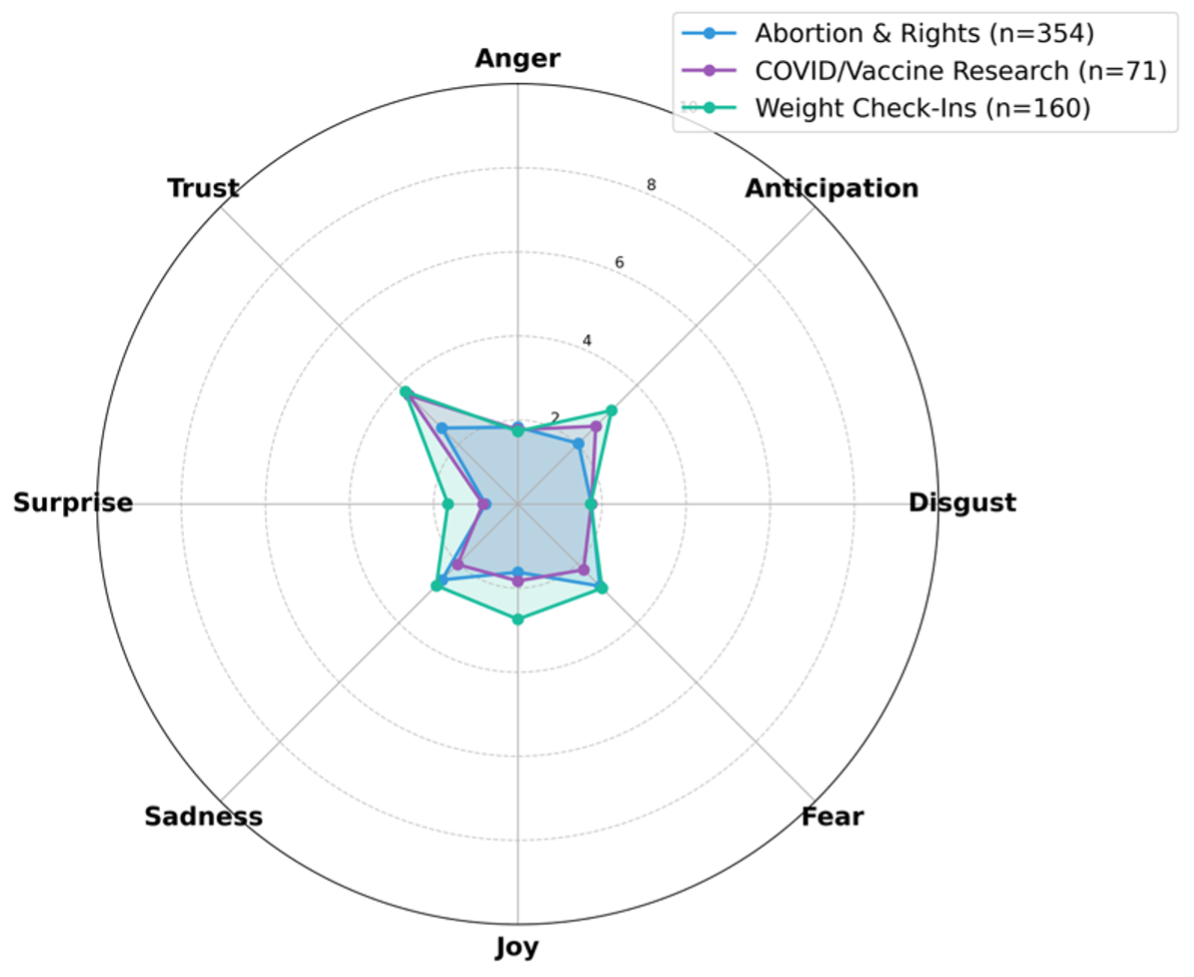
Low emotional intensity topics. Radar for Abortion & Rights (n=354), COVID/Vaccine Research (n=71), and Weight Check-Ins (n=160). Abortion & Rights and COVID/Vaccine Research are relatively flat; Weight Check-Ins is higher on Joy/Trust.

**Figure 4.**
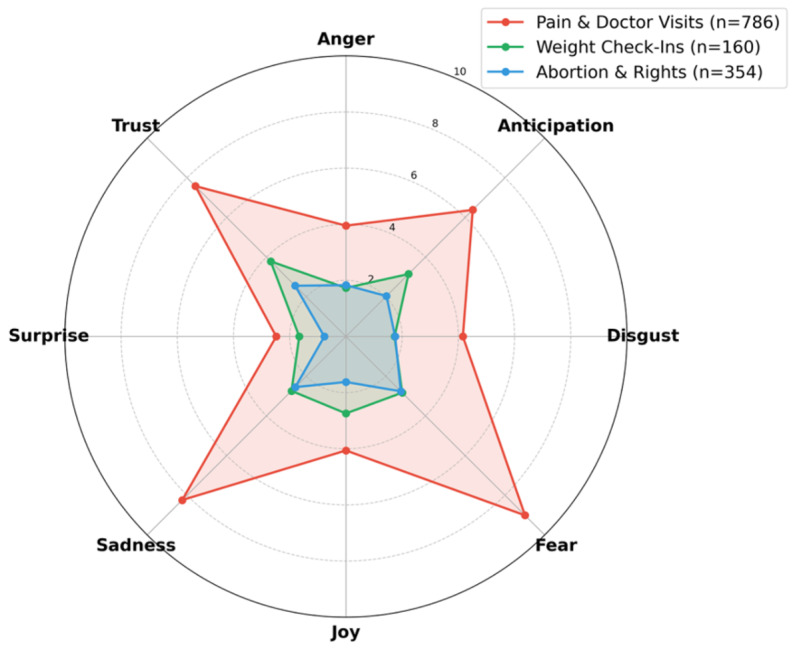
Emotional signatures comparison (contrasting topics). Direct comparison of Pain & Doctor Visits, Weight Check-Ins, and Abortion & Rights highlights stratification: Pain & Doctor Visits highest on Fear/Sadness, and Weight Check-Ins highest on Joy/Trust.

Table 3 details the distribution of 8 basic emotions across the identified topics. Analysis of specific emotion categories reveals that topics centered on advice-seeking, such as “Doctor & BC Discussions” and “PCOS & Fertility,” registered the highest scores for Trust (7.48% and 5.56%, respectively). Additionally, elevated levels of Joy were observed in topics such as “Sex & Pregnancy” (5.07%) and “Weight Check-Ins” (2.74%). These variations in emotional distribution are visualized in the radar plots in Figures 2-4.

**Table 3.** Topic-wise emotion frequencies (percentage of total words tagged per emotion) mean emotional content by topic, normalized by post count.

Topic name	Anger	Anticipation	Disgust	Fear	Joy	Sadness	Surprise	Trust
PCOS^a^ & Weight/Body	3.96	5.76	4.62	6.17	4.96	6.42	3.12	6.62
Endometriosis	1.56	2.63	1.44	2.54	2.00	2.35	1.09	3.38
PCOS Diet & Insulin	3.85	6.77	5.26	5.85	5.51	6.49	3.55	7.28
Pain & Doctor Visits	3.95	6.38	4.16	9.01	4.06	8.24	2.48	7.58
Daily Life (Endo)	2.83	3.86	2.43	4.78	3.17	4.77	1.59	4.49
COVID/Vaccine Research	1.77	2.62	1.75	2.21	1.83	2.03	0.83	3.66
Men/Women Interactions	3.46	5.27	2.81	4.21	4.42	4.08	2.24	6.05
Birth Control Methods	2.88	5.88	3.06	5.47	4.05	4.80	1.92	5.96
Doctor & BC^b^ Discussions	2.68	6.06	2.31	6.16	4.48	4.03	1.67	7.48
PCOS & Healthcare Access	2.19	3.05	1.78	2.91	2.24	2.71	1.09	3.70
Sex & Pregnancy	2.80	6.55	2.91	4.83	5.07	3.91	1.65	6.54
Abortion & Rights	1.83	2.04	1.75	2.76	1.62	2.55	0.77	2.56
Weight Check-Ins	1.73	3.15	1.73	2.83	2.74	2.74	1.66	3.79
PCOS & Fertility	2.48	5.25	2.85	4.05	4.01	4.20	2.41	5.56
Body Image & Hair	2.37	3.89	2.20	2.97	3.40	3.02	1.31	4.16

a PCOS: polycystic ovary syndrome.

b BC: birth control.

Figure 2 illustrates the emotional profiles of high-intensity topics. Consistent with the findings in RQ1, “Pain & Doctor Visits” exhibited the highest levels of “Fear” (9.01%) and “Sadness” (8.24%) in the dataset. These scores quantitatively align with the qualitative focus on distress regarding clinical interactions. In contrast, PCOS-related topics (“PCOS & Weight/Body” and “PCOS Diet & Insulin”) displayed a distinct emotional profile characterized by elevated “Anger” and “Disgust” alongside high “Trust” scores (reaching 7.28% for “PCOS Diet & Insulin”). This pattern indicates that expressions of frustration regarding the condition frequently co-occur with markers of community reliance.

Figure 3 displays the emotional profiles of topics with lower overall intensity. The “Abortion & Rights” topic exhibited a comparatively flattened emotional signature, with intensity scores remaining low across all 8 emotion categories (eg, Anger: 1.83%, Fear: 2.76%). This profile contrasts with the high-intensity patterns observed in clinical encounter topics. Unlike the highly expressive signatures found in “Pain & Doctor Visits,” the emotional profile for “Abortion & Rights” lacks distinct peaks in high-arousal emotions, despite the sensitive nature of the subject matter.

Figure 4 provides a direct comparison of the emotional profiles for “Pain & Doctor Visits” and “Weight Check-Ins.” The radar plot for “Pain & Doctor Visits” is characterized by peak intensities in “Fear” (9.01%) and “Sadness” (8.24%). In contrast, the profile for “Weight Check-Ins” is clustered around “Joy” (2.74%) and “Trust” (3.79%), with notably lower scores in negative emotion categories. This comparison illustrates the distinct emotional functions of these topics, contrasting threads focused on distress with those centered on positive reinforcement.

### RQ3: Changes Across the COVID-19 Period

**Figure 5.**
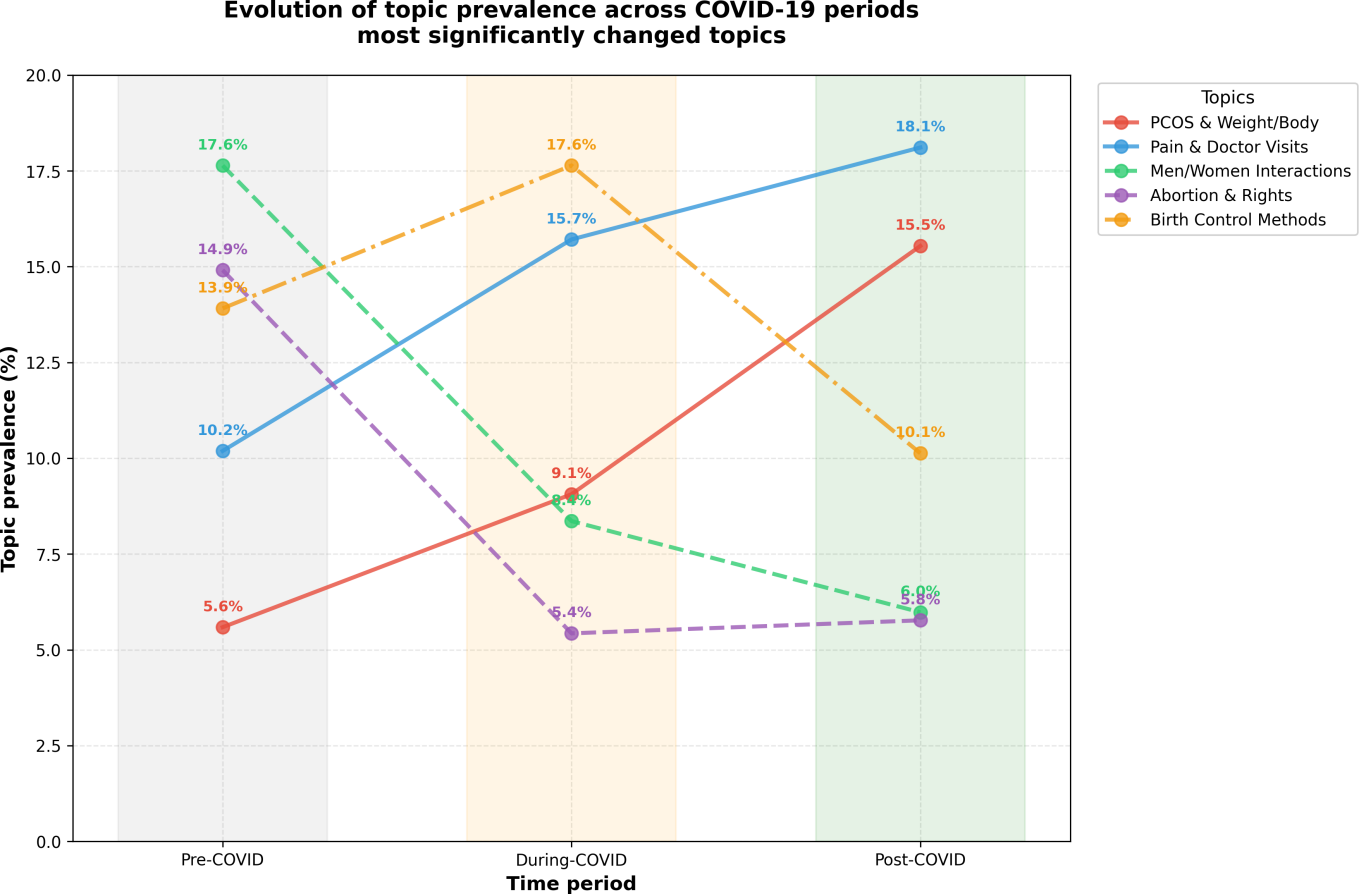
Evolution of topic prevalence across pre–, during, and post–COVID-19 periods for the 5 most shifted topics. PCOS & Weight/Body nearly triples (5.59% → 15.54%), Pain & Doctor Visits increases substantially (10.19% → 18.11%), while Men/Women Interactions and Abortion & Rights decline (17.64% → 5.98% and 14.91% → 5.77%, respectively). Birth Control Methods peaks during COVID-19 and tapers post–COVID-19 periods. Shaded bands indicate the 3 analysis periods. PCOS: polycystic ovary syndrome.

Figure 5 and Table 4 illustrate significant shifts in topic prevalence across the pre–COVID-19, during COVID-19, and post–COVID-19 periods. Topics related to social and political discourse showed a marked decrease; for instance, “Men/Women Interactions” declined from 17.64% to 5.98%, and “Abortion & Rights” decreased from 14.91% to 5.77%. Concurrently, topics focused on chronic conditions and clinical encounters increased in frequency. The prevalence of “PCOS & Weight/Body” nearly tripled, rising from 5.59% to 15.54%, while “Pain & Doctor Visits” became the most dominant topic, increasing from 10.19% to 18.11%. Qualitative analysis indicates that users frequently linked these health changes to the pandemic context, as illustrated by the following post:

*Hi! I got COVID in 2022 and lost my period for 3 months...7+ months later I was diagnosed with PCOS & insulin resistance, and my periods have been irregular ever since COVID...*

**Table 4.** Topic distribution across time periods (pre–COVID-19, during COVID-19, and post–COVID-19)^a^.

Topic	Overall, %	Pre–COVID-19, %	During COVID-19, %	Post–COVID-19, %
PCOS^b^ & Weight/Body	10.97	5.59	9.06	15.54
Endometriosis Exper.	4.16	5.34	4.16	3.67
PCOS Diet & Insulin	2.88	2.36	2.32	3.78
Pain & Doctor Visits	15.74	10.19	15.71	18.11
Daily Life (Endo)	6.45	4.84	7.70	5.62
COVID/Vaccine Research	1.42	1.24	1.49	1.42
Men/Women Interactions	8.95	17.64	8.36	5.98
Birth Control Methods	14.17	13.91	17.64	10.13
Doctor & BC^c^ Discussions	4.06	4.72	3.85	4.04
PCOS & Healthcare Access	2.34	1.86	2.36	2.52
Sex & Pregnancy	7.59	4.60	7.40	9.08
Abortion & Rights	7.09	14.91	5.43	5.77
Weight Check-Ins	3.20	4.47	3.19	2.68
PCOS & Fertility	4.40	2.48	3.50	6.30
Body Image & Hair	6.57	5.84	7.83	5.35

a Each cell indicates the number of posts (out of 4995) in that period that were classified into the given topic. This shows how the focus of discussions shifted over time.

b PCOS: polycystic ovary syndrome.

c BC: birth control.

Table 5 details the changes in emotion scores across the 3 periods. The analysis reveals statistically significant increases in negative emotion categories during the pandemic era. Comparing pre–COVID-19 with post–COVID-19 data, “Sadness” scores increased by approximately 46% (3.70-5.39), “Disgust” by 41% (2.44-3.44), and “Anger” by 28% (2.53-3.25). Textual analysis suggests that these emotional shifts coincided with increased discussions regarding symptom exacerbation and challenges in accessing health care. One user described this experience:

*...I got the Covid booster and flu shot and within WEEKS I was having severe period symptoms that I’ve never had before...I’ve ended up in the ER twice and passed out from the pain*.

**Table 5.** Average NRC emotion scores per post, grouped by COVID-19 period (pre–COVID-19, during COVID-19, and post–COVID-19).

Emotion	Pre–COVID-19	During COVID-19	Post–COVID-19	ANOVA *F* test (*df*) (2, 4992)	*P* value
Anger	2.53	2.91	3.25	12.87	<.001
Anticipation	4.41	5.17	5.25	9.43	<.001
Disgust	2.44	2.92	3.44	14.76	<.001
Fear	4.16	5.15	5.72	17.31	<.001
Joy	3.41	3.90	3.95	5.82	.003
Sadness	3.70	4.78	5.39	18.46	<.001
Surprise	1.70	1.96	2.13	6.91	.001
Trust	5.01	5.74	5.96	8.27	<.001

Simultaneous with the rise in negative emotions, the analysis also indicated increases in positive emotion categories (Table 5). “Trust” scores rose from 5.01 in the pre–COVID-19 period to 5.96 in the post–COVID-19 period. Similarly, “Joy” scores increased from 3.41 to 3.95 over the same time frame. These data demonstrate a concurrent increase in both distress-related emotions and markers of community support during the pandemic period.

### RQ4: Cultural Sensitivity and Engagement Patterns

The qualitative analysis of culturally situated discourse examined 147 threads that were validated as containing explicit cultural or religious references (Table 6). Coding of the interaction dynamics within these threads revealed structured patterns of support rather than random commentary. The analysis identified 3 distinct modes of engagement: Affirmation, Information Scaffolding, and Intercultural Bridging.

**Table 6.** Validation and reliability for culturally situated coding (thread level; N=335 flagged threads).

Metric	Values, n	Notes
Total threads analyzed	335	Flagged by computational filter
Reviewer agreement (threads)	327	97.6% agreement after reconciliation protocol
Threads validated as cultural	147	43.9% of flagged threads
Cohen κ (development subsample)	0.952	Strong interrater reliability

As detailed in Table 7, the analysis of 360 comments within these validated threads quantified the distribution of these support types. “Affirmation” was the most prevalent category, accounting for 38.9% of coded comments. This was followed by “Information Scaffolding” at 31.7%. The category of “Intercultural Bridging” was observed less frequently, representing 3.6% of the total comments .

**Table 7.** Engagement types for culturally situated threads (comment level; N=360 coded comments).

Engagement type	Count	Comments, %	Brief definition (primary code)
Affirmation	140	38.9	Validates culturally specific experiences; expresses empathy or solidarity
Information Scaffolding	114	31.7	Culturally tailored advice or resources (eg, fasting-compatible diet and language-concordant care)
Intercultural Bridging	13	3.6	Respectful cross-cultural learning or allyship
Other	93	25.8	General tips or off-topic replies lacking cultural relevance

The “Affirmation” category (38.9%) consists of comments that explicitly acknowledge and validate the cultural context of the original poster. Qualitative coding indicates that these responses function to establish shared identity or validate specific cultural constraints. For instance, one user responded to a post regarding fasting and medication: “I’m going through this during Ramadan too—thank you for saying it out loud. You’re not alone.”

This type of engagement focuses on validating the visibility of the user’s specific cultural experience. “Information Scaffolding" (31.7%) refers to comments that provide actionable advice tailored to specific cultural or religious requirements. Unlike generic medical advice, these comments often bridge clinical recommendations with cultural feasibility. A representative example involves a user suggesting a specific resource to navigate modesty concerns: “...check the Islamic Medical Association directory—finding a female Muslim endocrinologist meant I didn’t have to explain fasting or modesty concerns.” This category demonstrates the community’s role in adapting general health information to fit the user’s lived context.

The final category, “Intercultural bridging” (3.6%), involves contributions from users outside the poster’s cultural background. These comments are characterized by expressions of learning or allyship rather than advice-giving. One comment illustrates this dynamic: “I’m not from the same cultural background as you, but this post really opened my eyes to challenges I hadn’t considered. Thank you for sharing—it’s helping me understand why some of my friends might be hesitant to discuss certain health topics. Is there anything allies like me can do to be more supportive?” While numerically rare, these interactions mark instances of cross-cultural exchange and knowledge transfer within the threads.

## Discussion

### Principal Results

Our mixed methods analysis of 4995 posts and more than 460,000 comments portrays women’s health forums as far more than static information repositories or simple question and answer boards. Instead, our findings reveal a complex, adaptive sociotechnical infrastructure that actively compensates for deep-seated failures in formal health care systems. By mapping our results directly to our 4 RQs, we interpret how topic prevalence, emotional stratification, temporal shifts, and culturally situated exchanges intersect to reveal the community’s true function.

The topic modeling (LDA) results fundamentally challenge the assumption that online health communities are primarily drivers of “health literacy” or factual information seeking. While neutral, information-forward topics such as “Doctor & BC Discussions” existed, they were not the center of gravity. Instead, the discourse was dominated by systemic friction. The most prevalent topic, “Pain & Doctor Visits” (n=786), accounted for nearly 16% of the dataset. The lexical signature of this topic—dominated by terms such as “told,” “said,” and “doctor”—points to a structural breakdown in the clinical experience. We interpret this dominance as the forum functioning as a “shadow clinical space”: a venue where users perform the labor that the clinic failed to support. Here, users rehearse the medical visits they wish they had, validate experiences of dismissal (“medical gaslighting”), and crowdsource care trajectories that were missing in traditional settings. The sustained salience of “PCOS & Weight/Body” (n=548) further reinforces this mismatch; it reveals a patient population forced to manage chronic, complex conditions through peer deliberation because episodic clinical care is insufficient to address their daily lived reality.

Our sentiment and emotion analysis uncovered a distinct “emotional geography” within the forum, characterized by a sharp stratification between “territories of pain” and “clusters of agency.” We found that not all health struggles share the same emotional vocabulary. The “Pain & Doctor Visits” topic was not just negative; it was the “accumulated emotional burden” of the community, registering the lowest compound sentiment score (−0.2285) and saturated with the highest levels of Fear (9.01%) and Sadness (8.24%). This specific emotional profile signals that the distress is institutional—born from the trauma of invalidation—rather than purely somatic. In powerful contrast, topics centered on self-management and peer validation, such as “Weight Check-Ins,” registered the highest positive scores (+0.2633) and were clustered around Joy and Trust. This dichotomy confirms that the community is resilient enough to simultaneously hold space for the profound trauma of clinical dismissal and the celebration of personal control, effectively modulating its emotional affordances based on the user’s need.

The temporal analysis of the COVID-19 period identified a critical shift in the community’s function, which we term the “turn inward.” As the pandemic unfolded, discourse shifted significantly away from broader social and political topics (eg, “Men/Women Interactions” declined from 17.6% to 6.0%) and toward immediate, somatic struggles (eg, “PCOS & Weight/Body” nearly tripled in prevalence). Crucially, this shift revealed an “emotional paradox” that defines the community’s resilience. While the pandemic triggered a predictable and statistically significant surge in distress—with “Sadness” scores rising by 46% and “Fear” by 37%—this was accompanied by a simultaneous rise in “Trust” (from 5.01 to 5.96) and “Joy.” This counterintuitive finding suggests that the forum did not merely absorb the world’s pain; it acted as an engine of collective resilience. As external institutions destabilized and access to care became “fraught with risk,” the internal reliability of the peer network increased, validating the forum’s role as a sanctuary when traditional systems failed.

Finally, our qualitative analysis of culturally situated discourse identified a sophisticated, unwritten “protocol for care” that governs how the community navigates the intersection of health and identity. We found that effective support in these threads is not random but follows a normative “Affirmation-Scaffolding-Bridging” (ASB) sequence. Affirmation (38.9% of comments) serves as the necessary first step, establishing psychological safety by explicitly naming and validating the cultural frame (eg, “during Ramadan” and “modesty concerns”). This is followed by Information Scaffolding (31.7%), where users translate generic medical advice into culturally compatible action (eg, finding a female endocrinologist). The rare but transformative Intercultural Bridging (3.6%) then extends this support into broader community learning. This model demonstrates that in diverse health communities, identity validation is not just “nice to have”—it is a structural prerequisite for actionable advice. Systems that attempt to provide “facts” without first establishing this cultural safety risk reproducing the very frictions users come online to resolve. The ASB model advances beyond existing frameworks of online peer support in several important ways. Cutrona and Suhr’s [4] canonical Social Support Behavior Code categorizes support into informational, emotional, esteem, tangible, and network types—a taxonomy that treats these forms as concurrent and interchangeable, without prescribing any ordering among them. Our findings reveal, by contrast, that in culturally heterogeneous health communities, support provision is not merely categorical but sequentially ordered: emotional Affirmation must precede informational Scaffolding, which in turn enables cross-cultural Bridging. This staged architecture complements Walther’s [13] social information processing perspective, which established that online communicators build relational understanding through accumulated text-based cues over time—by demonstrating that in identity-sensitive health contexts, this relational groundwork cannot simply accumulate passively but must be actively and explicitly constructed as a prerequisite before instrumental support becomes actionable. Furthermore, while prior work has richly documented *what* types of support users exchange in online health communities [8,14], these frameworks largely treat cultural background as contextual rather than as a constitutive force that actively shapes the structure of the support interaction itself. Our model reframes culture as the organizing axis of the entire support sequence, advancing the field toward a more intersectional design agenda for digital health platforms.

### Comparison With Prior Work

This research sits at the intersection of HCI, computer-supported cooperative work, and health informatics. We build on three strands of prior work: (1) online platforms as peer-support infrastructures for health, (2) computational approaches to modeling and measuring health discourse at scale, and (3) intersectional design perspectives that account for emotion, culture, and temporality. Together, these literatures motivate our focus on how sociotechnical systems can more effectively support women’s health. While existing literature has established these platforms as vital “peer-support infrastructures” for information and connection, our findings compel a critical reframing of this role. We extend this foundational work by demonstrating that women’s health forums function not merely as spaces for supplemental support but as a *shadow clinical infrastructure* that actively compensates for systemic failures in formal health care. By integrating emotional, temporal, and cultural lenses, we move beyond characterizing *how* users support each other to explaining *why* they are driven to these platforms—often to perform the diagnostic and emotional labor displaced by the clinic.

Digital platforms have emerged as vital ecosystems for women to seek information, share lived experiences, and find emotional and peer support—especially where clinical systems fall short. Reddit, for instance, functions as a semianonymous infrastructure that enables candid discourse on stigmatized women’s health topics such as PCOS, endometriosis, and reproductive health [5,15]. This anonymity—paired with asynchronous communication—reduces the risk of judgment and encourages self-disclosure, facilitating emotional support and fostering a sense of belonging [15,16]. We extend these insights by characterizing this anonymous infrastructure as a “accumulated emotional burden.” Our results indicate that users leverage this safety not just for belonging but to perform the diagnostic and emotional labor displaced by the clinic—rehearsing medical scripts, validating dismissed symptoms, and comparing treatment protocols. Here, anonymity does not just reduce judgment; it neutralizes the power dynamics of the examination room, transforming the platform into a necessary mechanism for navigating structural health care deficits.

Emotional expression in these communities plays a central role in maintaining supportive dynamics. Expressive writing and self-disclosure help participants navigate emotional complexity by externalizing distress, validating experiences, and forming narrative meaning [17,18]. In peer-support forums, narratives infused with emotion—grief, relief, and humor—encourage empathy and promote group cohesion, reinforcing the role of these platforms as affective care infrastructures [14,17]. Similarly, studies of Alzheimer disease and related dementias caregiver communities show that Reddit fosters more prominent emotional support-seeking than traditional platforms such as ALZConnected, underscoring the value of anonymity and peer validation in shaping engagement [12]. Our analysis advances this understanding by mapping the distinct “emotional geography” of this engagement. We found that emotional expression is not uniform but highly stratified: topics such as “Pain & Doctor Visits” function as repositories for the community’s “accumulated emotional burden,” saturated with Fear and Sadness that reflect systemic trauma rather than simple distress. Furthermore, our temporal analysis reveals an “emotional paradox” during the COVID-19 pandemic. Contrary to the expectation that crisis simply breeds negativity, we observed a simultaneous surge in negative emotions (Fear and Sadness) and positive community markers (Trust and Joy). This suggests that high-emotion narratives do not just “encourage empathy” [14,17]; they actively metabolize external shocks, converting shared distress into collective resilience.

Moreover, these platforms operate less like static message boards and more like sociotechnical ecosystems of care. Moderation, community norms, and peer validation coalesce to create safe environments where vital conversations can unfold. For example, moderated mental health forums have demonstrated increased user engagement, candid emotional disclosure, and healthier linguistic coordination—outcomes indicative of trust and communal resilience [3]. We extend this view by operationalizing the specific “protocol for care” that sustains this resilience in culturally diverse contexts. Our findings suggest that this safety is not maintained by moderation alone but by a user-driven ASB sequence. Affirmation acts as the structural prerequisite, validating the user’s cultural reality before advice is offered. Furthermore, while “Intercultural Bridging” appears rare (3.6%), we argue that these interactions are theoretically significant: they represent the “breakthrough” moments where the community successfully leverages its established safety to dismantle stigma across cultural lines, transforming the forum from a simple support group into a dynamic engine of cultural competence.

Prior studies have leveraged sentiment analysis and qualitative coding to explore emotional communication in peer-support forums; these approaches have proven crucial in revealing how users articulate distress, empathy, and resilience through narrative exchanges [17,18]. Despite a growing body of work using topic modeling, misinformation detection, and sentiment classification, these efforts often remain siloed and lack integrated, nuanced insights into women’s health conversations. We address this fragmentation by integrating these computational lenses with a culturally situated qualitative analysis. Rather than treating topics, emotions, and cultural markers as separate signals, our study demonstrates their profound interdependence—showing, for instance, how specific clinical topics (“Pain & Doctor Visits”) do not just cluster words but actively concentrate specific emotional states (Fear and Sadness) and necessitate distinct cultural support protocols (Affirmation). This holistic approach allows us to move beyond simple classification to uncover the complex “emotional and cultural logic” that drives platform engagement.

Computational methods such as topic modeling have been instrumental in identifying the dominant themes across health forums—from anxiety and access to care to stigma and self-advocacy—by surfacing latent topics at scale [10,19]. Sentiment analysis provides a scalable means of assessing emotional valence and tone across large datasets, enabling researchers to track shifts in user sentiment during crises or thematic spikes [9,19]. For instance, Nwaoha et al [10] used longitudinal topic modeling on Reddit threads to trace evolving mental health discussions from pre– to post–COVID-19 periods, while Melton et al [19] combined topic modeling with sentiment analysis to monitor public response to COVID-19 vaccinations on Reddit. Tools such as VADER [20] make these analyses accessible and robust for social media data. We adapt these computational frameworks to the specific context of women’s health, using them to quantify the community’s structural resilience. While previous longitudinal studies [12] have mapped the trajectory of mental health symptoms, our analysis reveals a functional reorganization of the community itself. Our identification of the “Turn Inward”—a mass migration from political to somatic topics—demonstrates how these communities actively adapt their collective attention to survive systemic disruptions, offering a new lens on how health forums evolve during crises.

*Emotion lexicons*, particularly the NRC Emotion Lexicon, allow researchers to go beyond simple positive-negative classifications by labeling text with discrete emotional states such as fear, trust, or joy [21]. This enables finer-grained emotional mapping critical for understanding nuanced emotional responses in health discourse [2]. While powerful, lexicons such as NRC also face limitations—such as lacking support for emojis [22] or having issues with certain context-dependent words [1]. Despite these constraints, applying this granular lens to women’s health discourse proved essential. By moving beyond binary sentiment, we were able to detect the “emotional paradox” described in our principal results—specifically, the simultaneous rise of *Trust* and *Fear* during the pandemic. A simple polarity analysis would likely have averaged these conflicting signals into a neutral score, obscuring the complex reality that the community became *more* trusted even as the lived experience of its members became *more* distressing.

These computational approaches provide invaluable frameworks for analyzing emergent patterns in health-related social media discussions. However, most prior work remains domain-specific—focused on mental health, vaccination, or misinformation—and overlooks broader women’s health domains such as reproductive care, menstruation, and chronic gynecological conditions. Our study builds on this established HCI method tradition, applying *LDA for topic modeling*, *VADER for sentiment analysis*, and the *NRC Emotion Lexicon for emotion classification*, all embedded within a *temporal framework*. To achieve a more holistic understanding, we additionally layer in *qualitative cultural discourse analysis*, enabling richer, context-sensitive insights. Despite growing attention to individual aspects such as emotion, temporality, or culture, existing work largely treats these dimensions in isolation—leaving key intersectional questions unanswered.

The COVID-19 pandemic underscores the critical role of temporality in shaping digital health discourse. For example, longitudinal analyses of Reddit mental health communities show heightened discussion volume and user support at the pandemic’s onset, followed by declines that suggest emotional fatigue and shifting needs over time [23]. Additionally, symptom narrative analyses on Reddit reveal how discussion topics evolve over the course of individual illness episodes, mapping emotional dynamics in relation to real-world health progression [11]. Our analysis shows a strategic reallocation of attention: as the pandemic rendered external health care inaccessible, the community pivoted en masse from political or social topics to immediate somatic management (eg, PCOS and Pain). This suggests that in times of crisis, these forums do not just ’react’ with increased volume but structurally reorganize to serve as a survival mechanism.

Likewise, cultural and societal norms profoundly mediate how health information is discussed and received online. Religious values, familial expectations, and taboo influence narratives—determining both willingness to share and the nature of engagement. Culturally insensitive responses can deter participation and erode trust, especially among marginalized communities. Socioeconomic disparities—particularly in education and digital literacy—further compound this challenge, as they shape tone, access, and participation in health discussions [6]. Cross-cultural HCI studies have demonstrated clear differences in mental health expressions across global contexts, revealing that cultural identity shapes narrative style, emotional expression, and the perceived legitimacy of support [24].

Together, these insights illustrate that temporality matters—and culture matters. Yet, the literature still lacks a holistic, empirical account of how emotion**,** temporality, and culture intersect within women’s health communities to shape support-seeking, discourse dynamics, and platform engagement. Our study addresses this critical gap by delivering a mixed methods, temporally anchored, and culturally sensitive analysis—pointing toward design principles that can sustain adaptive, equitable, and emotionally responsive digital health ecosystems.

While our analysis was conducted on Reddit—a decentralized platform not built specifically for health—our findings are *formative* for the design of *new, dedicated* sociotechnical systems. The following implications are therefore proposed not as changes for Reddit itself but as an evidence-based framework for *future* health platforms that aim to be culturally responsive and resilient.

1. Design for navigating clinical failures:

- Clinical encounter tools: when posts rehearse or debrief visits, the platform can meet users where they are.
- Provide encounter-ready scaffolds: agenda builders that transform symptom narratives into question prompts, encounter logs that structure what happened and what to monitor, evidence snippets tied to community-endorsed resources, and post visit reflection cards that detect unresolved concerns (eg, persistent fear or anger signals) and suggest next steps.

2. Scaffolding cultural discourse:

- Community-curated and situated resources: affirmation clears space; scaffolding makes action possible. Introduce culture tags at thread or resource level (eg, fasting and modesty), with curated resource cards that surface within a thread when relevant terms appear (eg, directories of culturally sensitive clinicians). Add allyship prompts that nudge respectful questions for outsiders without burdening posters (eg, inline microetiquette and “ask-to-learn” templates).

3. Adaptive interfaces for collective emotional resilience: topics such as chronic pain carry heavy fear or sadness; progress-oriented threads concentrate joy or trust. Interfaces should modulate accordingly. Provide gentle modes in high-distress threads (eg, de-emphasize confrontational language and highlight validation first), strengthen positive feedback in progress threads (eg, low-friction celebration affordances), and pace attention through check-in prompts when sustained negativity suggests overload.

### Limitations

Several limitations must be considered when interpreting these findings. First, as noted in our “Methods” section, our study is not an exhaustive analysis of all “women’s health” on Reddit but a deep dive into 5 specific communities. Our sampling notably excludes major communities related to neurodivergence (eg, r/adhdwomen, r/autisminwomen), pregnancy, and miscarriage. Consequently, our broad topical and temporal findings (eg, the shift in topics during the COVID-19 period) are exploratory and characterize this specific ecosystem but should not be generalized to the entire platform.

Furthermore, our “top 1000 posts” sampling strategy biases our dataset toward high-visibility content and may not capture the full spectrum of less-upvoted or more niche discussions. This selection bias has a specific implication for the generalizability of the ASB model. Because top-ranked posts are by definition those that have attracted high community engagement, they may disproportionately represent interactions where the community’s support protocols worked well—threads that were collectively upvoted precisely because they demonstrated effective, affirming, and culturally responsive exchanges. It is therefore plausible that the 3-stage ASB sequence is less consistently observed in lower-engagement threads, where community norms may be less enforced, responses may be fewer or less considered, or where cultural framing may not receive the same validation. Future research should deliberately sample from a broader range of upvote distributions—including median- and low-visibility posts—to test whether the ASB model holds across the full engagement spectrum, or whether it emerges primarily in high-salience community interactions.

Methodologically, our reliance on the NRC Emotion Lexicon presents specific constraints in a health context. While standard in the field, lexicon-based approaches may misclassify clinical terminology; words such as “surgery,” “bleeding,” or “pain” may be tagged as “Fear” or “Sadness” even when used in a neutral, descriptive context. This potential for context collapse suggests that the high negative emotion scores in clinical topics (eg, “Pain & Doctor Visits”) should be interpreted as a signal of thematic intensity rather than purely psychological distress. Future work could mitigate this by using context-aware large language models to validate lexicon scores.

Finally, regarding our cultural analysis, the “Inter-Cultural Bridging” category represented only 3.6% of the coded comments. While theoretically significant, this low frequency limits our ability to statistically characterize the drivers of cross-cultural allyship. Future research should specifically oversample for these interactions to better understand the conditions that facilitate them.

### Conclusions

This study reframes online women’s health forums from supplemental support groups into essential shadow clinical infrastructures that actively compensate for the failures of formal health care. By integrating computational analysis with qualitative depth, we demonstrated that these communities do not merely host discussions; they perform the critical diagnostic and emotional labor—the “shadow work”—that is frequently displaced by clinical and cultural frictions. The “Turn Inward” observed during the pandemic and the accompanying “emotional paradox” reveal these platforms as adaptive engines of resilience, capable of restructuring their collective attention to function as sanctuaries when external institutions destabilize.

Furthermore, our identification of the ASB model challenges the HCI field to move beyond designing for simple information retrieval. We show that in culturally situated health contexts, “psychological safety” is not an intangible outcome but a structural prerequisite for effective advice-giving. Future digital health systems must therefore be designed not just to deliver facts but to operationalize these unwritten protocols of care—validating identity before offering intervention. Ultimately, this research provides an evidence-based framework for building sociotechnical systems that are as emotionally responsive and culturally competent as the communities they aim to serve.

## Supplementary material

10.2196/87782 Multimedia Appendix 1 Qualitative codebook for culturally situated discourse.

10.2196/87782 Checklist 1 GRAMMS (Good Reporting of A Mixed Methods Study) checklist for mixed methods studies.

## Abbreviations

API: Application Programming Interface
ASB: Affirmation-Scaffolding-Bridging
HCI: human-computer interaction
IRB: institutional review board
LDA: Latent Dirichlet Allocation
NRC: NRC Emotion Lexicon
PCOS: polycystic ovary syndrome
RQ: research question
VADER: Valence Aware Dictionary for Sentiment Reasoning
