# Supplementary material for: Peer Support in Online Women’s Health Communities: Mixed Methods Formative Analysis of Reddit Discourse
Source: JMIR Form Res. 2026 May 4;10:e87782. doi: 10.2196/87782 (PMC13138790; doi:10.2196/87782)
Supplement: Multimedia Appendix 1 [file formative-v10-e87782-s001.docx]

**Multimedia Appendix 1**

**Study:** A Formative Analysis of Emotion, Temporality, and Culture in Women's Health Forums **Coders:** Human Annotators (2) **Reliability:** Cohen’s κ = 0.952 (Development Subsample); Negotiated agreement for full dataset.

**Part 1: Thread-Level Inclusion Criteria.**

*Goal: Determine if a thread is "Culturally-Situated" and eligible for engagement analysis.*

| **Category** | **Definition & Criteria** |
| --- | --- |
| **Culturally-Situated Post** | A post that explicitly connects a women's health issue to a specific cultural, religious, or ethnic context.    **Inclusion Criteria (Must meet BOTH):**    1. **Explicit Reference:** The post contains direct mentions of cultural, religious, or ethnic identities/contexts (e.g., "Muslim," "Asian," "traditional family," "modesty").    2. **Health Connection:** The cultural context is explicitly linked to the health experience (e.g., taboos affecting care-seeking, religious fasting impacting medication, family norms influencing contraception). |
| **Exclusion (Not Culturally-Situated)** | **Incidental Mentions:** The post mentions a nationality or identity marker but it has no bearing on the health narrative (e.g., "I am travelling to [Country]..." or "I am [Nationality]..." without further context linking it to the health issue). |

**Part 2: Comment-Level Engagement Codes.**

*Goal: Characterize the primary mode of support offered in the top 10 comments of validated threads.*

| **Code** | **Definition** | **Inclusion Criteria (When to apply)** | **Examples (from dataset)** |
| --- | --- | --- | --- |
| **Affirmation** | **Validation of Identity & Safety.**    Comments that explicitly validate the user's specific cultural reality or emotional burden. | • Must acknowledge the cultural frame (not just generic "I'm sorry").    • Focuses on shared identity, solidarity, or validating that the user's cultural struggle is real/legitimate. | *"I’m going through this during Ramadan too—thank you for saying it out loud. You’re not alone."* ^1^ |
| **Information Scaffolding** | **Culturally-Compatible Action.**    Comments that provide specific advice, resources, or tactics tailored to the user's cultural constraints. | • Translates generic medical advice into a form compatible with the user's beliefs/norms.    • Suggests specific directories, doctors, or workarounds (e.g., non-gelatin meds, female-only clinics). | *"...check the Islamic Medical Association directory—finding a female Muslim endocrinologist meant I didn’t have to explain fasting..."* ^2^ |
| **Inter-Cultural Bridging** | **Cross-Cultural Allyship.**    Comments from users *outside* the poster's culture that express a desire to learn or support without overstepping. | • Commenter explicitly identifies as an outsider/ally.    • Asks respectful questions to understand the cultural nuance.    • Expresses gratitude for learning about a new perspective. | *"I'm not from the same cultural background... but this post really opened my eyes... Is there anything allies like me can do?"* ^3^ |
| **Other** | **Generic or Off-Topic.**    Comments that provide support but lack specific cultural relevance. | • Generic medical advice (e.g., "Take ibuprofen").    • Generic emotional support (e.g., "Hope you feel better") without cultural markers.    • Off-topic discussions. | N/A |
