# Supplementary material for: Peer Support in Online Women’s Health Communities: Mixed Methods Formative Analysis of Reddit Discourse
Source: JMIR Form Res. 2026 May 4;10:e87782. doi: 10.2196/87782 (PMC13138790; doi:10.2196/87782)
Supplement: Checklist 1 [file formative-v10-e87782-s002.docx]

GRAMMS (Good Reporting of A Mixed Methods Study) checklist.

| **Guideline Section** | **Description / Justification in Manuscript** |
| --- | --- |
| **Describe the justification for using a mixed methods approach to the research question** | **Section: Methods (Identifying and Analyzing Culturally-Situated Discourse)**    We justify the approach by stating that the hybrid design reflects a tradition of blending large-scale computational methods with qualitative depth to unpack the nuances of online health discourse. This approach yields a defensible basis for quantifying engagement patterns (scale) while preserving the contextual nuance (depth) necessary to inform culturally responsive design. |
| **Describe the design in terms of the purpose, priority and sequence of methods** | **Section: Methods (Identifying and Analyzing Culturally-Situated Discourse)**    We describe a sequential exploratory design consisting of two distinct phases. Phase 1 (Computational Filtering) was prioritized for recall to create a broad candidate pool. Phase 2 (Human Qualitative Thematic Analysis) followed to validate cultural situatedness and characterize engagement patterns. |
| **Describe each method in terms of sampling, data collection and analysis** | **Section: Methods (Data Collection; Topic Modeling; Sentiment Analysis; Human Qualitative Thematic Analysis)**    **Sampling/Collection:** We collected the top 1,000 posts from five specific subreddits using the Reddit API (PRAW), resulting in n=4,995 posts .    **Quant Analysis:** We employed Latent Dirichlet Allocation (LDA) for topic modeling (k=15) , VADER for sentiment polarity , and the NRC Emotion Lexicon for emotion classification .    **Qual Analysis:** We used a structured, two-step coding process with two independent coders, assessing inter-rater reliability (Cohen’s kappa = 0.952) and using a "negotiated agreement" protocol for the final dataset . |
| **Describe where integration has occurred, how it has occurred and who has participated in it** | **Section: Methods (Phase 1: Computational Filtering); Discussion**    Integration occurred at the sampling interface ("connecting"). The computational analysis filtered the massive dataset to identify a specific subset (n=335) containing cultural markers. This output became the direct input for the qualitative phase. Integration also occurred during interpretation, where quantitative emotional signatures were contextualized by qualitative narrative analysis. |
| **Describe any limitation of one method associated with the present of the other method** | **Section: Limitations**    We acknowledge that the computational reliance on the NRC Emotion Lexicon may lead to "context collapse" where clinical terms (e.g., "surgery") are misclassified as negative emotions . Additionally, the "top 1,000" sampling strategy, necessary for computational feasibility and relevance, biases the dataset toward high-visibility content, potentially excluding niche discussions that qualitative analysis might otherwise explore. |
| **Describe any insights gained from mixing or integrating methods** | **Section: Discussion (Principal Results; Comparison with Prior Work)**    Mixing methods allowed us to identify the "Emotional Paradox"—the simultaneous rise of Trust and Fear during COVID-19—which a simple polarity analysis would have obscured. Furthermore, integrating topic modeling with qualitative discourse analysis allowed us to characterize the forum not just as an information source, but as a "shadow clinical infrastructure," linking topic prevalence (Quant) to the specific labor of validating dismissed symptoms (Qual). |
